# Evaluation of Different Criteria in the Diagnosis of Left Ventricular Hypertrophy by Electrocardiogram in Comparison With Echocardiogram

**DOI:** 10.7759/cureus.26376

**Published:** 2022-06-27

**Authors:** João Pedro Marcato, Felipe Senra Santos, André Gama Palone, Gustavo Lenci Marques

**Affiliations:** 1 Internal Medicine, Federal University of Paraná, Curitiba, BRA; 2 Cardiology, Heart Institute, Clinical Hospital, Faculty of Medicine, University of São Paulo, São Paulo, BRA; 3 Medicine, Pontifical Catholic University of Paraná, Curitiba, BRA

**Keywords:** echocardiogram (echo), electrocardiography (ecg), left ventricular hypertrophy (lvh), cardiovascular, diagnostic techniques

## Abstract

Left ventricular hypertrophy (LVH) is an adaptive mechanism of the cardiac muscle due to increased activity demand or functional overload. The echocardiogram (ECHO) presents a better performance in relation to the electrocardiogram (ECG) for the diagnosis of LVH. However, the ECG is a low-cost and easy-to-reproduce diagnostic alternative and can be useful in services and locations where the ECHO is not yet easily accessible.

Recently, a new criterion for LVH was proposed by Peguero-Lo Presti. The aim of this article was to evaluate the electrocardiographic criteria for the diagnosis of LVH (Sokolow-Lyon, Cornell voltage, Romhilt-Estes, and Peguero-Lo Presti) in comparison to the diagnosis made by the ECHO.

ECHO and ECG from 142 patients' medical records were analyzed. Patients were divided into three groups according to the ECHO - control, eccentric LVH, and concentric LVH. Sensitivity, specificity, PPV, NPV, and accuracy of the four electrocardiographic criteria were evaluated in three scenarios - (1) LVH vs control, (2) concentric LVH vs control, and (3) eccentric LVH vs control.

Of the 142 patients included in the study, 100 (70.4%) had LVH. According to the type of hypertrophy, the 100 patients with LVH were divided into two groups - 41 (28.8%) had eccentric LVH and 59 (41.5%) had concentric LVH.

Of all the scenarios, the Peguero-Lo Presti criteria obtained the best sensitivity (1, 41%; 2, 33,9%; 3, 51,2%) and accuracy (1, 56,3%; 2, 58,4%; 3, 69,8%). The Sokolow-Lyon criteria showed greater specificity in all analyses (100%). None of the electrocardiographic criteria obtained sensitivity values ​​that would justify the use of the electrocardiogram as a screening test for LVH. No differences were found for eccentric and concentric LVH in terms of diagnosis using electrocardiographic criteria. We recommend the use of these criteria to confirm the diagnosis of LVH, especially in low-complexity services that do not have image-based diagnostic tests.

## Introduction

Left ventricular hypertrophy (LVH) consists of an adaptive mechanism of the cardiac muscle due to an increased activity demand or functional overload [1,2]. In general, the situations that trigger this response are increased pressure or volume load, increased metabolic requirement or high-output conditions, and genetic inheritance [1,3,4]. Hypertrophies can be characterized as concentric and eccentric, whereas in concentric, there is an increase in wall thickness with a reduction in cavity diameter, and in eccentric, there is an increase in thickness and cavity diameter [2,5].

Currently, the echocardiogram (ECHO) is one of the tests used for the diagnosis of LVH through the estimation of measurements and ventricular mass [2]. ECG examination provides data on cardiac electrical activity, which may indicate LVH from a pattern of left ventricular overload through changes in the electrocardiographic tracing [6]. Although the performance of the echocardiogram is superior in LVH analysis, since it is an examination that evaluates the cardiac dimensions, the ECG presents itself as a complementary screening alternative that has low cost and wide dissemination [6].

To date, several criteria and indexes for the diagnosis of LVH by ECG have been elucidated. In current medical practice, the criteria of Sokolow-Lyon, Cornell voltage, and Romhilt-Estes are most used [7].

Recently, a new criterion for the diagnosis of LVH by ECG was proposed by Peguero-Lo Presti, which showed greater sensitivity and accuracy when compared to the other criteria used today [8].

This study aimed to evaluate the electrocardiographic criteria of Peguero-Lo Presti, Sokolow-Lyon, Cornell voltage, and Romhilt-Estes in the diagnosis of left ventricular hypertrophy, establishing sensitivity, specificity, positive predictive value, negative predictive value, and accuracy.

## Materials and methods

This is an accurate, analytical, observational, cross-sectional, and retrospective study, in which medical records, ECG, and ECHO reports of patients at the Hospital de Clínicas of the Federal University of Paraná (HC-UFPR) were analyzed. Such documents were provided by the Pulmonology and Cardiology Unit of the Hospital de Clínicas of the Federal University of Paraná, therefore the researchers were not responsible for its execution. The study was authorized by the Research Ethics Committee of HC-UFPR. The free and informed consent term was waived due to the impossibility of carrying out it because there was no contact between the researchers and the research participants. All patient data remains confidential, held by the researchers.

Study population 

A total of 142 patients were selected and were divided into three groups - (1) patients with eccentric left ventricular hypertrophy, (2) patients with concentric left ventricular hypertrophy, and (3) control group. The patients participating in the study were randomly selected from the HC-UFPR echocardiography service database. Then the medical records of these patients were analyzed in search of an ECG. The inclusion criteria for the study were HC-UFPR patients who had undergone an ECG up to three months before or after the ECHO. All patients selected for the study have already reached adulthood, 20 years old, as proposed by the World Health Organization (WHO). Patients were excluded from the study if they had left ventricular hypertrophy due to congenital diseases, deposition diseases (such as amyloidosis and Fabry Disease), traces that prevent the analysis of LVH (such as left bundle branch block), medical records with significantly incomplete information for the study, and patients under the age of 20 years.

Electrocardiographic evaluation

The ECG was performed according to the international convention - graph paper, thermosensitive, machine speed of 25 mm/s, and amplitude of 10 mm/mV, including 12 leads (aVF, aVR, aVL, DI, DII, DIII, V1, V2, V3, V4, V5, and V6). 

The ECG of all 142 patients was analyzed, calculating the electrocardiographic criteria for the diagnosis of left ventricular hypertrophy. The electrocardiographic diagnostic criteria used were (1) Peguero-Lo Presti criterion (deeper S + SV4 ≥ 2.3 mV in women and ≥ 2.8 mV in men) [8]; (2) Sokolow-Lyon criterion (SV1 + RV5 or V6 ≥ 35 mV) [9]; (3) Cornell voltage criterion (RaVL + SV3 ≥ 20 mV for women and ≥ 28 mV for men) [10]; (4) Romhilt-Estes criterion, which allows the diagnosis of left ventricular hypertrophy through several different parameters to which points are assigned, the diagnosis being confirmed from five points, and for four points, the diagnosis is probable (increased amplitude of the R or S waves ≥ 20 mV in the frontal plane, or ≥ 30 mV in the horizontal plane - three points; alteration of ST-T in the absence of digitalis - three points; left atrial overload, Morris index, defined as a negative final component of the p-wave in V1 ≥0.04 s, and amplitude ≥ 1 mV - three points; deviation of the SAQRS axis to the left, in addition to -30° - two points; extended QRS ≥ 0.09 s without branch block pattern - one point; ventricular activation time ≥ 0.05 s in V5 and V6 - one point; alteration of ST-T in the presence of digital - one point) [11].

Echocardiographic evaluation

On the ECHO, the following parameters were evaluated - left ventricular diastolic diameter (LVDD), left ventricular systolic diameter, interventricular septum (ES) thickness, posterior wall thickness (EPP), cardiac mass, and relative wall thickness. Such data are used to identify the geometric pattern of the left ventricle using the formulas proposed by Devereux et al. [12]. 

LV mass (g) = 0.8 (1.04 {(ES + EPP + LVDD) 3 - LVDD3}) + 0.6g

The cut-off value used to define LVH, based on the relationship between ventricular mass and body surface, was 95 g/m² for women and 115 g/m² for men [13]. All echocardiographic examinations performed by the service where the study took place are performed on machines of the Philips brand, model Affiniti 70 (Minas Gerais, Brazil: Philips Medical Systems Ltd).

For the classification of LVH patients between eccentric and concentric, the calculation of the relative wall thickness (RWT) was used: RWT = 2EPP/LVDD. RWT values ​​≥ 0.45 were classified as concentric left ventricular hypertrophy. ERP values ​​< 0.45 were classified as eccentric left ventricular hypertrophy.

Statistical analysis

In the descriptive analysis of the data referring to age, sex, values ​​of the electrocardiographic criteria, and the presence of LVH, the Shapiro-Wilk test was performed to assess the normality of the sample, and later the Mann-Whitney test. For each electrocardiographic criterion used in the study, sensitivity, specificity, positive predictive value (PPV), negative predictive value (NPV), and accuracy were analyzed in three situations - LVH vs control, LV concentric vs control, and LV eccentric vs control, through the software IBM SPSS Statistics v.20.0 (Armonk, NY: IBM Corp.).

## Results

Of the 142 patients included in the study, 100 (70.4%) had left ventricular hypertrophy and 42 (29.5%) did not, integrating the control group. Of the total, 81 were female (57%) and 61 male (43%), with a mean age of 64.3 ± 12.6 years. According to the type of hypertrophy, the 100 patients with LVH were divided into two groups - 41 (28.8%) had eccentric LVH and 59 (41.5%) had concentric LVH. Table 1 shows the distribution of gender and age in relation to the type of LVH. In all groups, age and sex distribution were similar. The same analyses, comparing the two LVH subgroups (eccentric or concentric), are shown in Table 2.

**Table 1:** Age and sex distribution (LVH group vs control group) LVH: left ventricular hypertrophy

		LVH group	Control group	p-Value
Age (years)		66.4 ± 11	60 ± 14.4	0.073
Sex	Male	39 (27.5%)	22 (15.5%)	0.720
	Female	55 (38.7%)	26 (18.3%)	
Left ventricular mass (g/m^2^)		137.1 ± 29.9	87.8 ± 18	0.010
Left ventricular diameter (mm)		52.1 ± 7.3	45.5 ± 4.3	0.000
Relative wall thickness (RWT)		0.44 ± 0.09	0.43 ± 0.08	0.249
Sokolow-Lyon (mm)		17.7 ± 9.5	17 ± 6.7	0.016
Cornell (mm)		18.9 ± 9.6	13 ± 5.5	0.018
Peguero-Lo Presti (mm)		23.6 ± 11.4	16.6 ± 7	0.002

**Table 2:** Age and sex distribution (eccentric LVH vs concentric LVH) LVH: left ventricular hypertrophy

		Eccentric LVH	Concentric LVH	p-Value
Age (years)		64.6 ± 10.5	67.8 ± 11.2	0.429
Sex	Male	14 (9.8%)	25 (17.6%)	0.486
	Female	26 (18.3%)	29 (20.4%)	
Left ventricular mass (g/m^2^)		141.4 ± 29.3	133.9 ± 30.2	0.517
Left ventricular diameter (mm)		57.8 ± 5.7	47.9 ± 5.2	0.366
Relative wall thickness (RWT)		0.35 ± 0.04	0.51 ± 0.06	0.036
Sokolow-Lyon (mm)		17.7 ± 10.1	17.7 ± 9.1	0.782
Cornell (mm)		20 ± 10.1	18 ± 9.1	0.863
Peguero-Lo Presti (mm)		25.7 ± 11.4	22.1 ± 11.2	0.688

The results for each electrocardiographic criterion for the three situations (LVH vs control, concentric LVH vs control, and eccentric LVH vs control) in relation to specificity, PPV, NPV, and accuracy are shown in Tables 3, 4, 5, respectively.

**Table 3:** Performance of criteria in the assessment of left ventricular hypertrophy LVH: left ventricular hypertrophy; PPV: positive predictive value; NPV: negative predictive value

	Sensibility (%)	Specificity (%)	PPV (%)	NPV (%)	Accuracy (%)
Sokolow-Lyon	6	100	100	30.8	33.8
Cornell	26	97.6	96.3	35.6	47.1
Romhilt-Estes	28	78.5	75.6	31.4	42.9
Peguero-Lo Presti	41	92.8	93.1	39.8	56.3

**Table 4:** Performance of criteria in the assessment of concentric left ventricular hypertrophy LVH: left ventricular hypertrophy; PPV: positive predictive value; NPV: negative predictive value

	Sensibility (%)	Specificity (%)	PPV (%)	NPV (%)	Accuracy (%)
Sokolow-Lyon	5	100	100	42.8	44.5
Cornell	22	97.6	92.8	47.1	53.4
Romhilt-Estes	22	78.5	59	41.7	45.5
Peguero-Lo Presti	33.9	92.8	86.9	50	58.4

**Table 5:** Performance of criteria in the assessment of eccentric left ventricular hypertrophy LVH: left ventricular hypertrophy; PPV: positive predictive value; NPV: negative predictive value

	Sensibility (%)	Specificity (%)	PPV (%)	NPV (%)	Accuracy (%)
Sokolow-Lyon	7.3	100	100	52.5	54.2
Cornell	31.7	95.2	86.6	58.8	63.8
Romhilt-Estes	36.5	73.8	57.6	54.3	55.4
Peguero-Lo Presti	51.2	88.1	80.7	64.9	69.8

The sensitivity values ​​of all criteria were higher in the analysis of eccentric LVH and lower in the analysis of concentric LVH. Regarding specificity, all criteria maintained the same values ​​in the analysis of LVH and concentric LVH. The Cornell, Romhilt-Estes and Peguero-Lo Presti criteria showed lower specificity rates in the analysis of eccentric LVH compared to other analyses.

The PPV values, with the exception of the Sokolow-Lyon criterion, which remained 100% in the three analyses, were higher in the analysis of LVH and lower in the analysis of eccentric LVH. The NPV values ​​for all criteria were higher for eccentric LVH and lower for LVH. Regarding accuracy, all criteria were shown to be higher in the analysis of eccentric LVH, and lower in the analysis of LVH.

Of all the analyses, the Peguero-Lo Presti criterion obtained the best sensitivity, accuracy, and NPV rates. The Sokolow-Lyon criterion showed greater specificity and PPV in all analyses.

## Discussion

In the literature, a series of similar studies have already compared several electrocardiographic criteria for the diagnosis of LVH. The results are often controversial. A possible explanation lies in the fact that the formula for calculating LVH through echocardiography has changed over the years, as well as the reference values ​​for defining this condition in men and women. Among the studies compared with the results of the present study, only Gasperin et al., Peguero et al., Ramchand et al., Okin et al., and Domingos et al. used the formula proposed by Devereux to calculate the left ventricular mass [6,8,10,12,14,15]. Only the studies by Peguero et al. and Ramchand et al. used the reference for the definition of LVH proposed by Lang et al. (95 g/m^2^ for women and 115 g/m^2^ for men) [8,10,13].

Sokolow-Lyon criterion

The specificity rates of the Sokolow-Lyon criterion found in our study were 100% in all analyses, in accordance with the study in which this criterion was presented. In the original study by Sokolow and Lyon, in 1949, patients with previously diagnosed heart disease, with greater ventricular mass and advanced age, were compared to young individuals with normal ventricular mass [16]. Other studies have had results similar to ours, reaching 100% specificity for the Sokolow-Lyon criterion, such as Casale et al. and Domingos et al. [15,17]. In the study by Gasperin et al., based on a Brazilian population, the specificity found for the Sokolow-Lyon criterion was 88.80% for women and 71.88% for men, significantly distant from the rates found in the present study [6]. The PPV rates for the Sokolow-Lyon criterion found for all analyses were 100%, in accordance with the specificity rates. In the analysis by Gasperin et al., the PPV rates found were 57.58% for women and 38.63% for men, significantly lower than our results [6]. In the study by Casale et al., the PPV rates for this criterion were, again, 100%, in accordance with the specificity found in the same study [16,18].

The Sokolow-Lyon criterion showed a higher sensitivity in the diagnosis of the eccentric pattern (7.32%), later in the diagnosis of LVH without pattern differentiation (6%), and finally, the concentric pattern (5%). The sensitivity of the criterion obtained in the original study was 32%, significantly higher than the rates found in our study in all analyses, as well as in the study by Romhilt and Estes (42.5%) [11]. Other studies similar to ours have found higher sensitivity rates, and the ones that came closest to our results were Ramchand et al. (14%) and Peguero et al. (17%) [8,10]. Regarding NPV rates, according to the Sokolow-Lyon criterion, the eccentric LVH analysis obtained the highest value (52.5%), followed by the concentric LVH analysis (42.86%), and finally, the LVH analysis without pattern differentiation (30.88%). Gasperin et al. found a rate of 77.62% in the analysis made in women and 80.23% in the analysis made in men [6]. In the study by Casale et al., the NPV rate found for this criterion was 55%, similar to that found in the analysis of eccentric LVH in our study [16]. Regarding the accuracy of the Sokolow-Lyon criterion, the highest rate occurred in the analysis of eccentric LVH (54.22%), followed by the analysis of concentric LVH (44.55%) and the analysis of LVH without pattern differentiation (33.8%). The accuracy rate found by Casale et al. in their study was 65%, higher than our results [16].

Cornell voltage criterion

In the present study, the Cornell voltage criterion showed greater sensitivity in the diagnosis of eccentric LVH (31.71%), followed by the diagnosis of LVH without pattern differentiation (26%), and finally, the diagnosis of concentric LVH (22.03%), as what occurred with the Sokolow-Lyon criterion. In the original study by Casale et al., priority was given to the development of a criterion that was not dependent on the prevalence and severity of LVH, such as what occurred with the previously established criteria, and thus could present acceptable performance in diverse populations, including establishing distinct values ​​for the sexes [17]. The sensitivity for this criterion obtained in the original study, when prospectively analyzing 129 individuals (57 men and 72 women), was 41%. The sensitivity rate found by Okin et al. for this criterion was 22% [14]. The specificity of the Cornell criterion remained constant in the analysis of LVH without pattern differentiation and concentric LVH (97.62%). In the analysis of eccentric LVH, specificity was slightly lower (95.24%). In the original study, this criterion had a specificity of 98%, similar to that found in our study [16]. In the study by Okin et al., the specificity was 87%, lower than that found in our study [14]. In our study, the PPV rates of the Cornell criterion were higher in the analysis of LVH without pattern differentiation (96.3%), later in the analysis of concentric LVH (92.86%), and finally, in the analysis of LVH eccentric (86.67%). The PPV in the original study was 91% [16]. The decreasing order in NPV rates follows eccentric LVH (58.82%), concentric LVH (47.13%), and LVH without pattern differentiation (35.65%). Unlike the PPV, the NPV of the original study was found to be higher than that found in our study (73%) [16]. The accuracy achieved by the Cornell criterion in our study was 63.86% in the analysis of eccentric LVH, 53.47% in the analysis of concentric LVH, and 47.18% in the analysis of LVH without pattern differentiation. In the original study, the accuracy obtained was 76%, greater than those found in the present study [16].

Romhilt-Estes criterion

The sensitivity of the Romhilt-Estes criterion in our study was greater in the analysis of eccentric LVH (36.59%), followed by the sensitivity of LVH analysis without pattern differentiation (28%), and later, in the analysis of concentric LVH (22.03%). Both original studies by Romhilt and Estes were carried out from the autopsy of patients who had severe heart valve disease and hypertension, which probably caused a very pronounced LVH [11,18]. In addition, several patients had non-cardiac causes of death, such as cancer. These diseases can have cachexia, causing reduced cardiac mass, as well as electrocardiographic voltage [16]. Such characteristics may explain the sensitivity rate of 60% found in the original studies [11,18]. In the study by Casale et al., the sensitivity of the Romhilt-Estes criterion in the evaluation of 414 patients (175 men with a mean age of 49 ± 18 years; 239 women with a mean age of 47 ± 19 years) was 19% [16]. The Romhilt-Estes criterion specificity showed a rate of 78.57% in the analyses of LVH without pattern differentiation and concentric LVH. In the analysis of eccentric LVH, the specificity was 73.81%. This value is lower than the result found in the studies in which this criterion was presented (96.8% and 97%) [11,18]. The specificity found by Casale et al. was 96% [16].

The PPV rates of the Romhilt-Estes criterion in this study were higher in the analysis of LVH without pattern differentiation (75.68%), followed by the analysis of concentric LVH (59.09%), and the analysis of eccentric LVH (57%, 69%). In the study by Casale et al., the PPV of the Romhilt-Estes criterion was 77%, higher than all of our analyses [16]. The highest NPV rate occurred in the analysis of eccentric LVH (54.39%), followed by the analysis of concentric LVH (41.77%) and, finally, the LVH analysis without pattern differentiation (31.43%). The NPV rate found by Casale et al. was 60%, similar to that found in the analysis of eccentric LVH in our study [16]. The accuracy achieved by the Romhilt-Estes criterion in our study was 55.42% in the analysis of eccentric LVH, 45.54% in the analysis of concentric LVH, and 42.96% in the analysis of LVH without pattern differentiation. Regarding the NPV rate, the value found for accuracy by Casale et al. (61%) is close to the value found in the analysis of eccentric LVH in our study [16].

Peguero-Lo Presti criterion

Unlike the traditional criteria, which are based on the analysis of the higher voltages of the R wave in several leads, the criterion proposed by Peguero-Lo Presti is composed only by the analysis of the S wave, which was more related to an increase in left ventricular mass. A possible explanation for this finding would be the fact that the vector generated by the depolarization of the free ventricular wall and the myocardium can be better represented by the final part of the QRS complex, which is the S wave [8]. 

The Peguero-Lo Presti criterion had the highest rates of sensitivity, NPV, and accuracy among all the criteria analyzed in our study. The highest value of the NPV rates occurred in the analysis of eccentric LVH (64.91%), followed by the analysis of concentric LVH (50%) and LVH without pattern differentiation (39.8%). The accuracy rates followed the same order, obtaining, respectively, 69.88%, 58.42%, and 56.34%.

The sensitivity rates of the Peguero-Lo Presti criterion in our study were 51.22% in the analysis of eccentric LVH, 41% in the analysis of LVH without pattern differentiation, and 33.9% in the analysis of concentric LVH. In the original study, the sensitivity of this criterion was significantly higher (62%) [8]. In the study by Ramchand et al., 138 patients with aortic stenosis and with a high prevalence of comorbidities, with a mean age of 74 years (± 11 years), 61% of whom were men, underwent echocardiography and ECG, in which the Peguero-Lo Presti criterion obtained a sensitivity rate of 49% [10]. In relation to specificity rates, the original study obtained a rate of 90%, very close to the rates obtained in the present study (92.86% in the analyses of LVH without pattern differentiation and concentric LVH, and 88.1% in the analysis of eccentric LVH) [8]. In the study by Ramchand et al., the specificity of the Peguero-Lo Presti criterion was 84% ​​[10]. Finally, the highest PPV values ​​for the Peguero-Lo Presti criterion occurred in the analysis of LVH without pattern differentiation (93.18%), followed by concentric LVH (86.96%) and eccentric LVH (80.77%).

In the study by Tavares et al., which included 592 patients aged over 70 years, the sensitivity of the Peguero-Lo Presti criterion was 51.9% [19]. Regarding specificity, this criterion obtained a performance of 82.1%. In the meta-analysis by Yu et al., which covered five studies, the sensitivity found for the Peguero-Lo Presti criterion was 52%, while the specificity was 85% [20]. The results of both studies were similar to the findings for eccentric LVH in our study.

Comparing the eccentric and concentric LVH analyses, there were no differences between the criteria that presented the best performances, since the Sokolow-Lyon criterion remained with the highest rates of PPV and specificity in both, in the same way that it occurred with the criterion of Peguero-Lo Presti, which showed better performance in NPV rates, sensitivity and accuracy in both analyses. In general, all criteria presented better performances in sensitivity, NPV, and accuracy in the analysis of eccentric LVH, while the specificity and PPV of all criteria had better performances in the analysis of concentric LVH.

Limitations

ECG is an examination that analyzes the cardiac electrical activity, unlike the ECHO which evaluates the cardiac mass. However, we understand that the ECG can be useful in services and locations where the echocardiogram is not yet easily accessible, serving as a useful tool for the diagnosis and follow-up of LVH, as well as for optimizing the use of more complex tests in a health system [19]. In order to reduce the limitation resulting from the different nature of the examinations, we chose not to include patients in which this factor could cause more discrepancies and alterations, as in the case of heart deposition diseases. 

Although magnetic resonance imaging (MRI) is the current gold standard for the assessment of left ventricular hypertrophy, we were not able to use it in our work, which is another limitation of the study [1]. However, ECHO is a faster and more portable tool, characteristics that contribute to being more used in clinical practice for the assessment of LVH, which is one of the reasons for choosing it for our work [1]. Furthermore, in the literature, the comparison between the ECG and the ECHO is a common methodology for the evaluation of electrocardiographic criteria in the diagnosis of LVH. Since the publication of the Peguero-Lo Presti criterion, several studies have been carried out in this way in order to evaluate its performance, including a meta-analysis, whose results were similar to those found in our work [19-27].

## Conclusions

In this study, we did not find differences for eccentric and concentric LVH in terms of diagnosis using electrocardiographic criteria. Among the criteria analyzed, Sokolow-Lyon presented the best performance in the specificity tests, although the criteria of Peguero-Lo Presti and Cornell presented similar performance. Regarding sensitivity and accuracy, the Peguero-Lo Presti criterion performed better. However, none of the electrocardiographic criteria obtained sensitivity values ​​that would justify the use of the electrocardiogram as a screening test for LVH. In patients with high pre-test probability of LVH, such as in hypertensive patients, the PPV of the Sokolow-Lyon, Cornell, and Peguero-Lo Presti criteria is high. Therefore, we recommend the use of these criteria to help confirm the diagnosis of LVH, especially in low-complexity services that do not have image-based diagnostic tests, such as ECHO and MRI. More studies are needed to corroborate the results and better guide the use of electrocardiographic criteria for the diagnosis of left ventricular hypertrophy in medical practice.
